# Subcutaneous tunnelling versus conventional insertion of peripherally inserted central catheters in hospitalized patients (TUNNEL-PICC): a study protocol for a randomized controlled trial

**DOI:** 10.1186/s13063-022-06682-2

**Published:** 2022-09-15

**Authors:** Yohan Kwon, Eung Tae Kim, Soo Buem Cho, Jae Hwan Lee, Dong Jae Shim

**Affiliations:** 1 Department of Radiology, Ajou University Hospital, Ajou University School of Medicine, Suwon-si, Gyeonggi-do Republic of Korea; 2 Department of Radiology, Hanyang University Guri Hospital, Hanyang University College of Medicine, Guri-si, Gyeonggi-do Republic of Korea; 3 Department of Radiology, Ewha Womans University Seoul Hospital, College of Medicine, Ewha Womans University, Seoul, Republic of Korea; 4 Department of Radiology, Seoul National University College of Medicine, Seoul National University Bundang Hospital, Sungnam-si, Gyeonggi-do Republic of Korea; 5 Department of Radiology, Incheon St. Mary’s Hospital, College of Medicine, The Catholic University of Korea, Seoul, Republic of Korea

**Keywords:** Peripherally inserted central venous catheter, Central venous catheterization, Central line-associated bloodstream infections, Catheter-related bloodstream infections

## Abstract

**Background:**

Peripherally inserted central catheters (PICCs) are now widely used in modern medicine, and associated complications have also increased. Central line-associated bloodstream infection (CLABSI) is the most serious complication because it can cause extended hospital stays and increase costs. Furthermore, it can contribute to dire consequences for critically ill patients. Subcutaneous tunnelling for central venous catheters is an accepted method to reduce the risk of CLABSI. However, it is not generally adopted for PICC placement in most hospitals because its safety and efficacy have not been thoroughly evaluated.

**Methods:**

In this multi-institutional, prospective, non-blinded pragmatic randomized controlled trial, 1694 patients treated at five referral hospitals will be assigned to one of two parallel arms (conventional and tunnelled PICC groups) using computer-generated stratified randomization. The conventional group will undergo PICC placement by routine practice. In the tunnelled PICC (tPICC) group, additional subcutaneous tunnelling will be applied. Patients will be followed until PICC removal or the end of this study. The primary endpoint is whether subcutaneous tunnelling reduced the rate of CLABSI compared to the conventional method. The secondary endpoints are technical success rates, complications including exit-site bleeding or infection, and the procedure time between the groups.

**Discussion:**

Subcutaneous tunnelling is a widely used method to reduce catheter-associated infection. However, it has not been thoroughly applied for PICC. A randomized trial is needed to objectively assess the effects of the subcutaneous tunnel in PICC placement. This TUNNEL-PICC trial will provide evidence for the effectiveness of subcutaneous tunnelling in decreasing the risk of CLABSI.

**Trial registration:**

Clinical Research Information Service (CRiS) KCT0005521

## Administrative information

Note: the numbers in curly brackets in this protocol refer to SPIRIT checklist item numbers. The order of items has been modified to group similar items (see http://www.equator-network.org/reporting-guidelines/spirit-2013-statement-defining-standard-protocol-items-for-clinical-trials/).

**Table:** 

Title {1}	Subcutaneous tunnelling versus conventional insertion of peripherally inserted central catheters in hospitalized patients (TUNNEL-PICC): A study protocol for a randomized controlled trial
Trial registration {2a and 2b}	Registry: Clinical Research Information Service (CRiS) (https://cris.nih.go.kr/cris) Identifier: KCT0005521 (https://cris.nih.go.kr/cris/search/detailSearch.do/19481) Date of Registry: 21 October 2020
Protocol version {3}	Ver. 1.4, Date: 16 June 2020
Funding {4}	The study is financially supported by Reyon Pharmaceutical Co. and Genoss Co., Ltd.
Author details {5a}	1. **Yohan Kwon, MD, PhD** whitetsm@hanmail.net Department of Radiology, Ajou University Hospital, Ajou University School of Medicine, Suwon-si, Gyeonggi-do, Republic of Korea 2. **Eung Tae Kim, MD, Ph.D.** ket9818@hanmail.net Department of Radiology, Hanyang University Guri Hospital, Hanyang University College of Medicine, Guri-si, Gyeonggi-do, Republic of Korea. 3. **Soo Buem Cho, MD, Ph.D.** sbcho@ewha.ac.kr Department of Radiology, Ewha Womans University Seoul Hospital, College of Medicine, Ewha Womans University, Seoul, Republic of Korea 4. **Jae Hwan Lee, MD, Ph.D.** lzhwanmd@gmail.com Department of Radiology, Seoul National University College of Medicine, Seoul National University Bundang Hospital, Sungnam-si, Gyeonggi-do, Republic of Korea. 5. **Dong Jae Shim, MD, Ph.D.** inharad@naver.com Department of Radiology, Incheon St. Mary’s Hospital, College of Medicine, The Catholic University of Korea, Seoul, Republic of Korea
Name and contact information for the trial sponsor {5b}	Reyon Pharmaceutical Co.: Cho Ja Hyun, jaycho@reyonpharm.co.kr, 8th floor, 416 Yeongdong-daero, Daechi-dong, Gangnam-gu, Seoul, Republic of Korea Genoss Co. Ltd: Ho young Bae, hybae@genoss.com, 1^st^ floor, Gyeonggi R&DB Center, 105, Gwanggyo-ro, Yeongtong-gu, Suwon-si, Gyeonggi-do, Republic of Korea
Role of sponsor {5c}	Reyon Pharmaceutical Co. and Genoss Co., Ltd supported this study with a grant. However, neither funder had roles in the study design, data collection, and analysis, publication decision, or manuscript preparation.

## Introduction

### Background and rationale {6a}

Intravenous catheterization plays a pivotal role in patient care in modern medicine. Over the past decade, the use of peripherally inserted central venous catheters (PICCs) has continuously increased due to their advantages over other central venous catheters. They can centrally infuse vesicant or irritant agents from safe peripheral access. They are also versatile, easy to insert, and carry a relatively low rate of infection [1, 2]. However, PICC-associated bloodstream infections have been reported at rates of 0.6–7.4% and are as frequent as non-tunnelled central venous catheters [1, 3–8]. Central line-associated bloodstream infection (CLABSI) often requires intravenous antibiotic therapy, prolonged hospitalization, and even mortality in critically ill patients [9]. Traditionally, subcutaneous tunnelling has been used for central venous catheter placement (i.e. cuffed-tunnelled haemodialysis catheter, Apheresis catheter, or implantable venous port) [10]. Although subcutaneous tunnelling effectively reduces catheter-related infection, it has not been frequently used in PICC insertion, except for paediatric central line placement [11]. In 2001, Selby et al. reported the technical feasibility and safety of subcutaneous tunnelling on PICC insertion [12]. In addition, a bi-centre retrospective study demonstrated the protective effect of subcutaneous tunnelling on PICC-associated CLABSI [13]. However, no randomized controlled trial has compared the impact of subcutaneous tunnelling-applied PICC (tPICC) versus conventional PICC (cPICC) insertion with a focus on CLABSI. We hypothesized that using the subcutaneous tunnel for PICC insertion would effectively reduce the infection rate, even without tunnel-dedicated devices. This multi-institutional, open-label, parallel-group, pragmatic, randomized controlled trial study is designed to compare the catheter-related bloodstream infection rates of tPICC and cPICC in hospitalized patients.

### Objectives {7}

The objective is to evaluate the effect of subcutaneous tunnelling in PICC placement on the rate of CLABSI.

### Trial design {8}

This trial will be a prospective, randomized, controlled, investigator-initiated, multi-institutional, open-blind superiority study in five referral hospitals in the Republic of Korea. The allocation will be a 1:1 ratio of two parallel groups.

## Methods: participants, interventions, and outcomes

### Study setting {9}

This multi-institutional randomized controlled trial will be performed in five hospitals located in the metropolitan city of Korea: (1) Ajou University Hospital, (2) Hanyang University Guri Hospital, (3) Ewha Women’s University Seoul Hospital, (4) Seoul National University Bundang Hospital, and (5) Incheon St. Mary’s Hospital, Catholic University of Korea. All five hospitals are regional referral and educational institutions with more than 530 beds and can provide services to in- and outpatients. In addition, Seoul National University Bundang Hospital is a public hospital, and private academic foundations run other hospitals.

### Eligibility criteria {10}

**Fig. 1:**
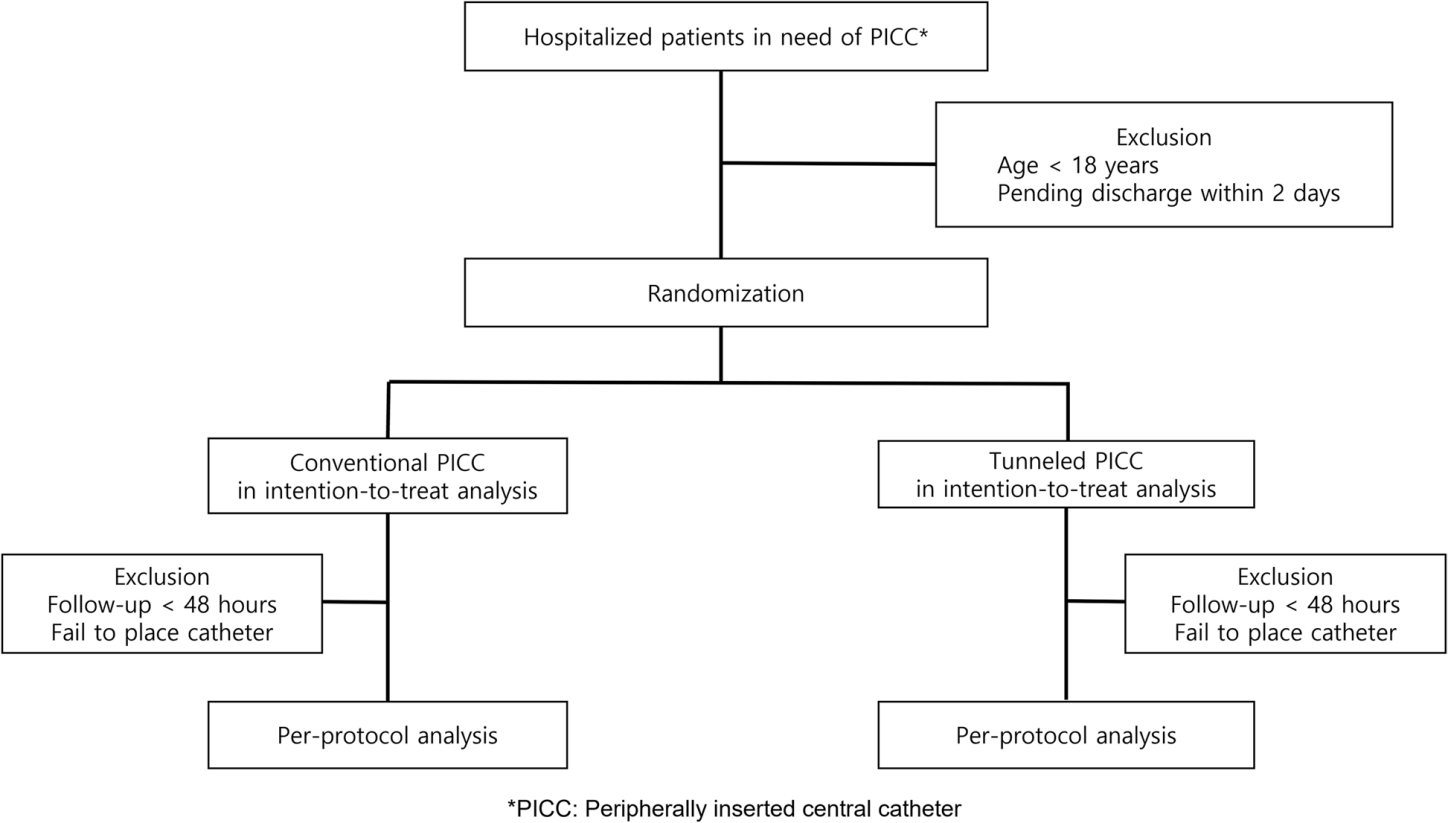
The CONSORT flow chart of this study

Eligible patients are >18 years of in-patients who require PICC insertion, according to the physician’s decision. Because the diagnosis of CLABSI requires at least 48 h of dwelling time, patients with pending discharge or transfer to another hospital within 2 days after catheterization will be excluded. In addition, this study will be pragmatic, and participants will not be excluded due to ongoing medical conditions, e.g. ongoing infection, malignancy, or immunity. Figure 1 shows the CONSORT flow chart of this study. Both tPICCs and cPICCs will be placed by one interventional radiologist at each institution designated for this study. Participants who will be treated on an outpatient base will be not included because infection-associated symptoms can be missed.

### Who will take informed consent? {26a}

Mostly, it has taken a single-working day from PICC requests to implementation in all five hospitals. Informed consent will be obtained by investigators of each institution at least 1 day before PICC placement. In patients with urgent medical necessity, which the referring physician will decide, informed consent will be obtained within at least 6 h of the procedure. Investigators will provide information sheets to participants and a detailed explanation of the study before obtaining informed consent.

### Additional consent provisions for collection and use of participant data and biological specimens {26b}

Not applicable.

### Interventions

#### The explanation for the choice of comparators {6b}

We hypothesize that using the subcutaneous tunnel for PICC insertion will reduce the CLABSI rate compared to conventional methods.

#### Intervention description {11a}

**Fig. 2:**
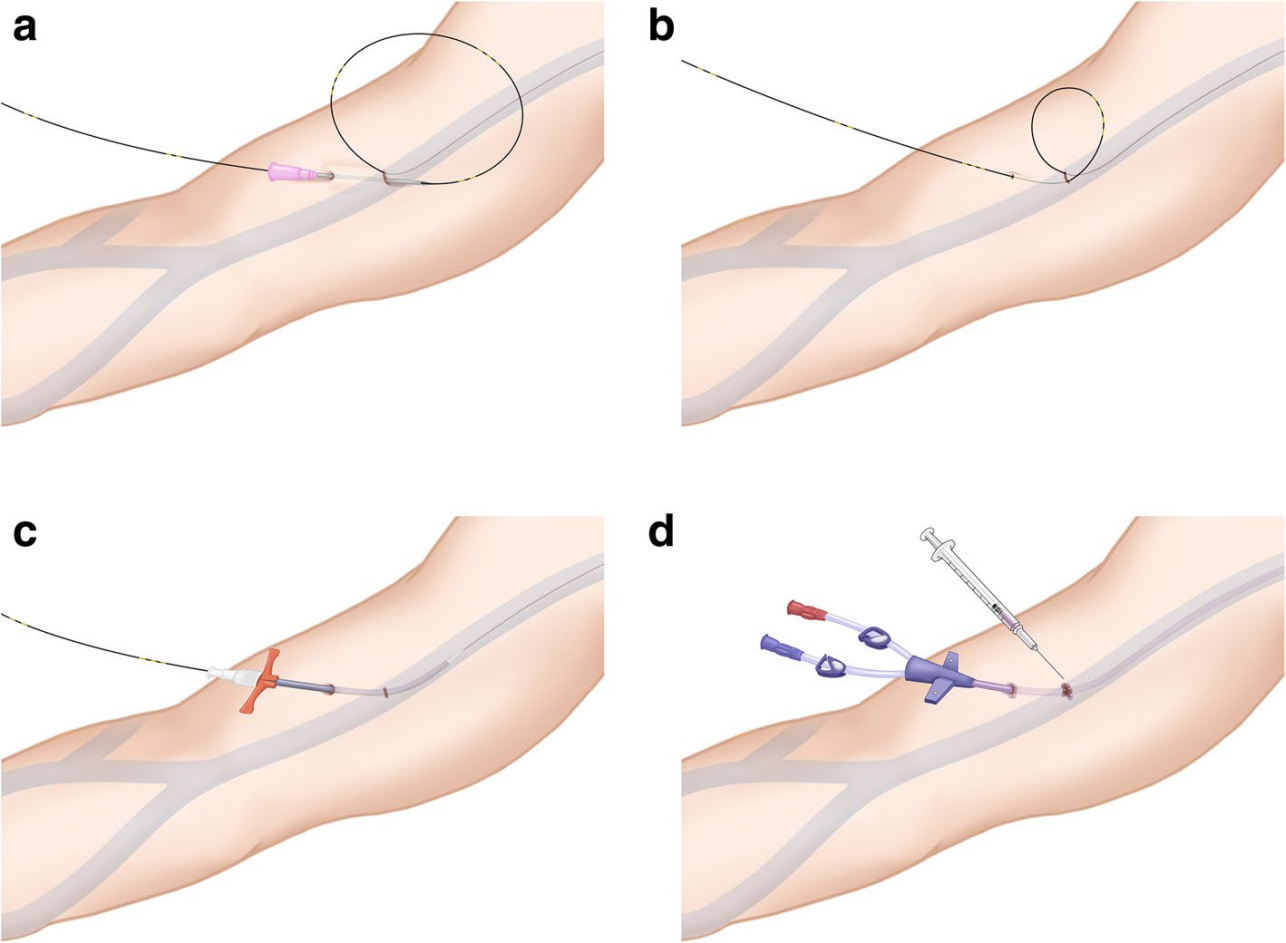
**A** Subcutaneous tunnel creation while placing a peripherally inserted central catheter (PICC). First, venepuncture under ultrasonography guidance will be performed with a puncture needle included in the PICC set, and a guidewire will be placed at an upper arm vein. Then, a subcutaneous tunnel will be created with an 18-gauge needle 1-inch away from the initial venepuncture site. **B** A guidewire will be passed through the needle under the subcutaneous tunnel. The loop will be resolved with a gentle snapping of the guidewire. **C** A peel-away sheath will be placed in the vein under the subcutaneous tunnel and over the guidewire. **D** Both wounds (initial venepuncture and catheter-exit sites) will be closed with glue (Histoacryl; B. Braun, Rubí, Spain)

Participants will be allocated into two groups. The cPICC group will have PICC placement with the traditional method under ultrasonography and fluoroscopic guidance in an angiography suite. Procedures will be implemented under hand hygiene, maximal sterile barrier, and chlorhexidine. The targeted arm will be sterilized with a mixture of chlorhexidine and isopropyl alcohol, and a sterile drape will be placed to cover the entire procedure field from head to toe [14]. A 5-French, dual lumen PICC from a vendor will be used (UNIS; Genoss Co., Gyeonggi-do, Korea). The tPICC group will undergo PICC placement in the same manner and place with additional subcutaneous tunnelling. Most commercially available PICCs contain no tunnellers or Dacron cuffs. Thus, after vein puncture with the access needle, a Nitinol guidewire will be placed as usual. We will make a tunnel 2–3 cm distal to the initial venepuncture site using an additional 18-gauge needle, and the guidewire will be retrogradely passed through the needle. After resolution of the loop over the venepuncture site, a peel-away sheath will be placed over the wire. The catheter will be trimmed to the distance between the venepuncture site and cavoatrial junction plus the subcutaneous tunnel before being inserted in the usual manner. The initial venepuncture and exit-site wounds will be closed by applying a small amount of *n*-butyl-2-cyanoacrylate (Fig. 2).

#### Criteria for discontinuing or modifying allocated interventions {11b}

Patients who do not want to participate in the trial or accidentally lose the PICC within 48 h after the procedure will be excluded from the study. There are no criteria for modifying allocated intervention.

#### Strategies to improve adherence to interventions {11c}

This study includes one-time intervention during the procedure and is a pragmatic study. All procedures will be performed by interventional radiologists involved in this study. Investigators will share procedure details on tunnelling methods by a standard manual and workshops

#### Relevant concomitant care permitted or prohibited during the trial {11d}

Not applicable. This is a pragmatic study. After PICC insertion, participants will be treated by their disease course. Relevant concomitant care will not be provided nor prohibited during trial.

#### Provisions for post-trial care {30}

Subcutaneous tunnelling is a widely used central venous catheter placement procedure with no serious reported complications. However, investigators will try to attenuate any damage from the intervention. When it is impossible to recover from irreversible injury, an insurance program for this study will provide compensation. Patients will not be compensated for their participation in the study.

#### Outcomes {12}

The primary outcome is the effect of subcutaneous tunnelling in PICC placement on the rate of CLABSI. We will use the National Healthcare Safety Network surveillance definition [15]. CLABSI will be defined as a laboratory-confirmed bloodstream infection where the PICC is in place for more than 48 h and must meet both of these criteria: participants have a recognized pathogen identified from one or more blood specimens by culture- or non-culture-based microbiologic test, and organisms identified in the blood are not related to an infection at another origin (e.g. mucosal-barrier injury). A board-certified infection disease specialist will diagnose CLABSI at each institution, independent of this study for blinding.

The secondary outcomes include rates of local infection and bleeding from exit sites, extra procedure time for subcutaneous tunnelling, and technical success, defined as the rate of successful catheter tip placement to the cavoatrial junction. The local infection will be defined as one or combined symptoms followings: insertion site swelling with tenderness, local heating sense, or pus. Bleeding from exit sites will be marked when PICC cannot be kept in place due to continuous bleeding. Minor oozing causing dressing change will not be counted as this outcome. We will divide technical success into two categories. If there is a failure in puncturing the target vein or placing the catheter to the cavoatrial junction, we will define it as ‘impossible PICC.’ In case of subcutaneous tunnelling failure after target vein puncture, we will explain it as ‘impossible tunnelling.’

### Participant timeline {13}

**Table:** 

Timepoint**	Study period			
	Enrolment	Allocation	Post-allocation	Close-out
	***− 1–3 days***	0	***Until catheter removal or hospital discharge***	***After catheter removal or hospital discharge***
**Enrolment:**				
**Eligibility screen**	X			
**Informed consent**	X			
**Demographic information**	X			
**Allocation**		X		
**Interventions:**				
***Conventional PICC***			X	
***Tunnelled PICC***			X	
**Assessments:**				
***Demographics***	X			
***Laboratory test***	X			
***Procedure details***			X	
***Procedure-associated complications***			X	
***Delayed complications***				X

### Sample size {14}

According to previous reports about PICC, infection rates range from 0.6 to 7.4% [1, 3–5]. In a previous retrospective study, the CLABSI rate of tPICC was 2.6% [13]. The sample size was calculated by assuming the same infection rate in this trial to verify the prior retrospective study result. In this trial, 2 × 677 participants are needed to prove a reduction of the infection rate from 6.2 to 2.6% for the primary outcome, at a two-sided α level of 0.05 and statistical power of 90%. A total of 1694 participants will be included in this study to account for a 20% dropout rate (PASS, version 16, NCSS statistical software). Data will be analysed on an intention-to-treat basis according to their originally assigned group.

### Recruitment {15}

According to the inclusion and exclusion criteria, all hospitalized patients requesting PICC will be potential candidates in this study. Physicians independent of this study will discuss the necessity of PICC with the participants. If the participants agree with catheterization, their physicians will consult with interventional radiology via an electronic medical record system. Investigators (interventional radiologists or clinical research coordinators) will review the participants’ medical records, and interventional radiologists will visit their wards to obtain informed consent. When obtaining informed consent, each candidate will have respectful discussions to ensure prudent decisions for the patients. A total of 339 patients will be enrolled from each of the five institutes by non-competitive recruitment.

### Assignment of interventions: allocation

#### Sequence generation {16a}

Randomization numbers will be generated by the R program (blockrand function in the package ‘blockrand’) using a 1:2 to 1:6 random block. An independent statistician generated multiple datasets, and an independent research organizer (Medsoft, Hwasung Gyeonggi, Korea) will apply one of the datasets to the electronic case report form (e-CRF) while blinded.

#### Concealment mechanism {16b}

Just before the procedure and after aseptic skin preparation, a circulating nurse who is not part of this trial will access the secure, password-protected, online-randomized database. The nurse will inform the investigator that the subject is in the cPICC or tPICC group.

#### Implementation {16c}

An independent statistician will create the computer-generated block randomization list. Then, each eligible participant with informed consent will be sent to the angiography suite and randomly assigned to a group immediately before the procedure by an independent circulating nurse in the angiography suite.

### Assignment of interventions: blinding

#### Who will be blinded {17a}

There is an unavoidable risk of bias in this type of randomized controlled trial where the intervention cannot be blinded to interventionists, participants, or care providers. However, this study has a single-blind characteristic due to following reasons. Participants can only presume their procedure on the grounds of their scar, but they cannot be assured about their group because procedures will be performed under aseptic drapes that screen the patient’s visual confirmation. It is difficult to know whether a tunnel is made or not because they are subcutaneous, and multiple skin incisions can also be made for conventional PICC. Furthermore, the interventionist will not confirm their group. As for care providers, mainly ward nurses can also presume the group but cannot be assured of the allocations for the same reasons as the participants. Outcome assessors will be blinded to allocation. The e-CRF on assignment will not be accessible to outcome assessors. Data analysts will be blinded until the end of the study.

#### Procedure for unblinding if needed {17b}

This study is unblinded.

### Data collection and management

#### Plans for assessment and collection of outcomes {18a}

In line with the pragmatic study, the participants will be treated for their condition according to the initial treatment plan. Daily physical examination and laboratory tests will be performed on a treatment schedule. Blood culture and laboratory testing will be performed as usual management when the participants have a symptom or sign of infection. Laboratory tests include white blood cell count with differentiation, erythrocyte sedimentation rate, and C-reactive protein level.

#### Plans to promote participant retention and complete follow-up {18b}

This study plans to enrol hospitalized patients, and adverse events (AEs) will be evaluated during hospitalization. We have no plan to promote retention.

#### Data management {19}

All data will be secured in an independent online server (Medsoft) outside hospitals. The investigators will be responsible for all data entry and management. At least two investigators will check all data.

#### Confidentiality {27}

All collected data will be coded with a code number, the only reference to participants’ identification during the study period to maintain anonymity. Informed consent forms will be secured in a locked cabinet in a password-locked secure place in each hospital. Data will be stored for 3 years after the end of this study according to the Enrollment Decree of the Bioethics and Safety Act of Korea. All data will be destroyed after that period, but data storage extension will be possible with Institutional Review Board (IRB) permission.

#### Plans for collection, laboratory evaluation, and storage of biological specimens for genetic or molecular analysis in this trial/future use {33}

Necessary laboratory blood tests and cultures will be performed depending on the clinical situation

### Statistical methods

#### Statistical methods for primary and secondary outcomes {20a}

Independent sample *t*-tests or Mann-Whitney *U* tests (continuous variables) and chi-square or Fisher’s exact tests (categorical variables) will be used to compare the two study groups. Data will be examined by cluster analysis, including the hospital level and ICU vs. general wards. The infection (CLABSI) rates will be reported as incidence per 1000-catheter dwelling days [16]. The rate ratio of both groups and 95% confidence interval and the *p*-value will be calculated by exact rate ratio test assuming Poisson counts. Cox proportional hazards regression will be used to estimate adjusted hazard ratios and 95% confidence intervals (CIs) for ‘time to infection.’ Hazards ratios with 95% CIs will be calculated for every complication. A *p*-value less than 0.05 will be considered significant.

#### Methods for additional analyses (e.g. subgroup analyses) {20b}

Subgroups will be divided according to their accompanying diseases, including diabetes mellitus, malignant tumour (or haematologic malignancy), immune insufficiency (human immunodeficiency virus infection or organ transplantation), end-stage renal disease, or comorbidity of more than one of those conditions.

#### Methods in analysis to handle protocol non-adherence and any statistical methods to handle missing data {20c}

Data produced following protocol non-adherence will not be included in the study and will be disclosed. An effort will be made to reduce missing data to a minimum. We will handle missing data with multiple imputations (MICE Package, R ver. 4.0.3, The R Foundation for Statistical Computing, Vienna, Austria). Missing values will be handled appropriately following guidelines [17]

#### Plans to give access to the complete protocol, participant-level data, and statistical code {31c}

The entire protocol will be available on the registry website and published here.

### Oversight and monitoring

#### Composition of the coordinating Centre and trial steering committee {5d}

The five authors (one from each institute) take full responsibility for scientific validity, study quality, study conduction, procedures, patient management after AEs, and quality of final study results and reports. All five authors share information through periodic meetings and discuss the study and appropriate management when problems occur.

#### Composition of the data monitoring committee, its role, and reporting structure {21a}

Each monitoring member will hold data monitoring in each institute. During monitoring, a data monitoring committee (DMC) will check whether the source document for the subject is appropriate, confirm that the consent acquisition process and storage are appropriate, review the overall trial performance, conduct an e-CRF review, review the researcher’s binder, collect any AEs from the subject, and follow. In addition, the committee is planning to monitor through verification of safety evaluation and data collection.

Any catheter-related AEs will be checked during the follow-up period and recorded at e-CRF. In case of more than grade II AEs, it will be reported to the principal investigator (DJ Shim) within 24 h. Each DMC member will judge whether the AE is associated with the procedure, and any related AE will be immediately reported to the IRB and principal investigator. Each researcher should report any severe AE (≥ grade III) and any other unexpected problems to the IRB within 15 days.

Three independent members will be appointed to the data and safety monitoring board (DSMB; Seungjae Lee [Department of Applied Bioengineering, Graduate School of Convergence Science and Technology, Seoul National University, Seoul, Korea], Young Seo Cho [Department of Radiology, Hanyang University Guri Hospital, Guri-si, Gyeonggi-do, Republic of Korea], Minuk Kim [Department of Radiology, Seoul Metropolitan Government-Seoul National University Boramae Medical Center]).

#### Adverse event reporting and harms {22}

AEs will be classified according to the Common Terminology Criteria for Adverse Events (CTCAE) guideline [18]. If more than three minor AEs (< grade III) occur at one site, the investigator of that site will report the AEs to the DSMB. In case of one severe AE (CTCAE III, IV, or V), a DSMB meeting will be held for safety evaluation. In addition, daily checks for each case will be carried out by the research team of each institution and will be communicated with the other research teams.

#### Frequency and plans for auditing trial conduct {23}

Patient monitoring by an independent monitor will occur until each new 200 cases are collected or every three months if cases do not reach 200. The inspection centre of each institution appointed inspectors to conduct systematic inspections of trial-related activities and documents. Patients and data evaluation will be performed independently of each institution’s investigators or the trial sponsors.

#### Interim analyses {21b}

Interim analyses will not be performed.

#### Plans for communicating significant protocol amendments to relevant parties (e.g. trial participants, ethical committees) {25}

Any change in this trial protocol will be reported to the IRB of each institute and trial registry.

#### Dissemination plans {31a}

The results of this study will be published in a peer-reviewed medical journal.

## Discussion

This TUNNEL-PICC trial will assess the effectiveness of subcutaneous tunnelling on PICC placement about the CLABSI rate. The results of this study will provide evidence for the application of subcutaneous tunnelling for PICC insertion and help reduce catheter-associated infection.

PICCs have been widely used in contemporary medical practice for their perceived safety, convenience, cost-effectiveness, and versatility. PICC is generally accepted as safe at insertion and relatively free from severe AEs such as CLABSI during the dwelling period [1]. However, recent reports indicated that the incidence of PICC complications is similar to that of standard central venous catheters [5]. This warrants a study adequately designed to verify the effectiveness of an additional subcutaneous tunnelling method over the conventional non-tunnelling method in reducing catheter-related infection.

Techniques and safety regarding subcutaneous tunnelling on PICC were documented by Selby and colleagues [12], but the infection rate in tPICC was not thoroughly evaluated. Kim et al. recently reported that subcutaneous tunnelling could reduce CLABSI in PICC with no significant increase in procedure time [13]. One randomized controlled study regarding tunnelling in patients who had undergone chemotherapy [19]. However, they assessed a relatively small number of participants (*n* = 129) and limited the study population to cancer patients. Thus, a prospective and randomized controlled study is needed to evaluate the effectiveness of subcutaneous tunnelling in PICC. This trial will be the first multicentre randomized controlled research to investigate whether tunnel-PICC insertion reduces catheter-related infection.

There are several limitations of this study. First, although participants and care providers cannot be sure of group allocation, they cannot be blinded. However, the outcome assessor will be blinded. Secondly, this study will be conducted in referral teaching hospitals of various sizes with different characteristics. These can contribute to a heterogeneous cohort but also reflects real-world practice.

This study will be the largest multi-institutional pragmatic randomized controlled trial that can provide guidelines for PICC insertion for patients who are vulnerable to infection.

## Trial status

At the time of manuscript submission, 700 patients have been recruited. Initial recruitment started on 16 November 2020. Recruitment and patient follow-up are still ongoing. The approximate date of recruitment completion is June 2023. This protocol is version 1.4 dated 16 June 2020.

## Data Availability

Datasets generated and/or analysed in this study will be available from the corresponding author on reasonable request after all identifiable information has been removed.

## Abbreviations

AE: Adverse event
CI: Confidence interval
CLABSI: Central line-associated bloodstream infection
cPICC: Conventional peripherally inserted central catheter
CTCAE: Common Terminology Criteria for Adverse Events
DMC: Data monitoring committee
DSMB: Data and safety monitoring board
e-CRF: Electronic case report form
PICC: Peripherally inserted central catheter
SVC: Superior vena cava
tPICC: Subcutaneous tunnelling-applied peripherally inserted central catheter
